# Merkel cell carcinoma metastasis and dermatofibrosarcoma protuberans presenting as a collision tumour: a case report and review of the literature

**DOI:** 10.4076/1752-1947-3-7493

**Published:** 2009-06-30

**Authors:** Daniel-Johannes Tilkorn, Marcus Lehnhardt, Jörg Hauser, Adrien Daigler, Heinz Homann, Hans Steinau, Cornelius Kuhnen

**Affiliations:** 1 Department of Plastic Surgery, Burn Center, Hand Center, Sarcoma Reference Center, BG-University-Hospital "Bergmannsheil", Ruhr-University Bochum, Buerkle-de-la Camp Platz, 44789 Bochum, Germany; 2 Institute of Pathology, BG-University-Hospital "Bergmannsheil", Ruhr-University Bochum, Buerkle-de-la Camp Platz, 44789 Bochum, Germany

## Abstract

**Introduction:**

Merkel cell carcinoma and dermatofibrosarcoma protuberans are two very rare neoplasms. The simultaneous occurrence of two different tumour entities at the same anatomical site, collision tumours, is a rare phenomenon.

**Case presentation:**

We present a rare case of a 74-year-old woman with a previous history of a recurrent dermatofibrosarcoma protuberans presenting with a metastatic Merkel cell carcinoma. Further investigation revealed a collision tumour of a metastatic lesion of the Merkel cell carcinoma within a tumour relapse of a dermatofibrosarcoma protuberans.

**Conclusion:**

Synchronous occurrence of two different tumour entities is extremely rare and has not been described for Merkel cell carcinoma and dermatofibrosarcoma. Merkel cell carcinoma, a tumour of the elderly or immunocompromised patients, leads to early metastasis and can be expected to be the limiting factor for prognoses.

## Introduction

Primary neuroendocrine tumour of the skin, also known as Merkel cell carcinoma (MCC), was first described by Toker in 1972. It is a rare but aggressive tumour entity of sun-exposed skin of the elderly in the age range of 60 to 90 years. The origin of this tumour is thought to be a line of differentiation according to Merkel cells. Under homeostatic conditions, the Merkel cells, discovered by Friedrich Merkel in 1875, are normal constituents of the basal layer of the epidermis and hair follicles. A higher density of Merkel cells is found in the glabrous epithelium of the digits, lips and oral cavity and hair bearing skin. They represent slow acting mechano-receptors with close contact to unmyelinated nervous fibres in the superficial dermis. Whether these cells are of a neuroendocrine or epidermal-ectodermal nature is still unclear.

MCC, with a female predominance, [1] has a predilection for the head and neck region, a potential for early metastasis and is rarely encountered on the extremities and trunk. Conditions leading to impaired immunity, such as immunosuppression therapy [2], human immunodeficiency virus infection and chronic lymphocytic leukaemia have been associated with a higher incidence of MCC.

Dermatofibrosarcoma protuberans (DFSP) is another uncommon skin malignancy. As an intermediate neoplasm it has a local aggressive growth pattern but it rarely metastasizes. In the uncommon event of metastasis they are often found in the lungs and in the lymph nodes [3]. A high recurrence rate complicates the course of the disease and requires wide local excision with clear margins.

Clinically, DFSP often presents as slow growing nodular masses of the skin on the trunk or proximal extremities.

Simultaneous occurrence of two different tumour entities at the same anatomical site is a very rare event. Cases of haematopoietic neoplasms in association with breast and skin malignancies have been reported. To our knowledge this is the first reported case of a synchronous occurrence (collision tumour) of MCC and DFSP.

## Case presentation

A 74-year-old, Caucasian woman from Germany with a long history of local recurrences of DFSP on her right upper limb was readmitted for surgical intervention of a tumour of her right axilla. On clinical examination she presented with two firm tumour masses in the right axilla and on the thoracic wall with a size of 6 × 7 cm and 6 × 5 cm. Both lesions displayed central ulceration. In addition, the patient requested the resection of a small (1.7 × 1.6 cm) nodular skin lesion of her right elbow region.

The DFSP of her right shoulder region was first diagnosed in 1991. Following several incomplete tumour resections and local progressive tumour growth she was transferred to our hospital in 2001. Tumour resection was carried out and radiation therapy was suggested but refused by the patient. Despite clear resection margins the tumour recurred locally 10 months later and, at this stage, the tumour had infiltrated the brachial plexus. Since then, additional surgical interventions for local recurrences of the DFSP were required every 5 to 9 months. Fifteen years after the initial diagnosis of the DFSP, at readmission for a local recurrence, the tumour extended from the axilla along the thoracic wall to the lateral aspect of the ipsilateral scapular and an additional small (less than 2 cm) reddish blue subcutaneous nodular lesion was observed at the dorsal aspect of the right elbow.

**Figure 1:**
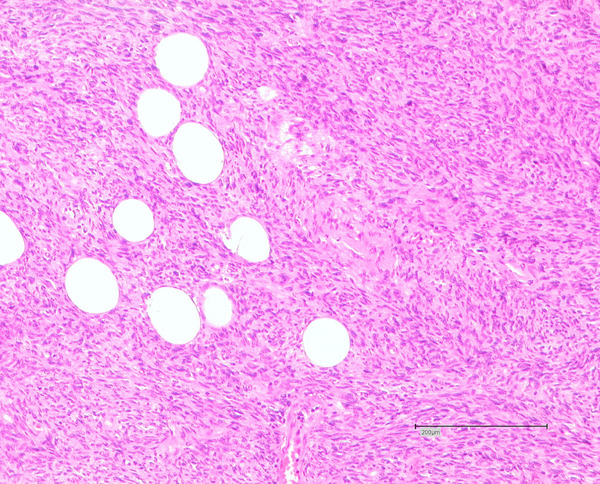
**Haematoxylin and eosin stain of the axillary tumour**. Note the characteristic partial storiform, partial fascicular, growth of the dermatofibrosarcoma protuberans.

Histologically the two tumour masses in the axilla and thoracic wall confirmed the clinical diagnosis of a local recurrence of the known DFSP. The tumours were highly cellular with partly ovoid, partly spindle shaped nuclei with small fascicular cell formations. Due to these clear detectable fascicular areas, these recurrences were classified as DFSP with fibrosarcomatous transformation (Figure 1).

**Figure 2:**
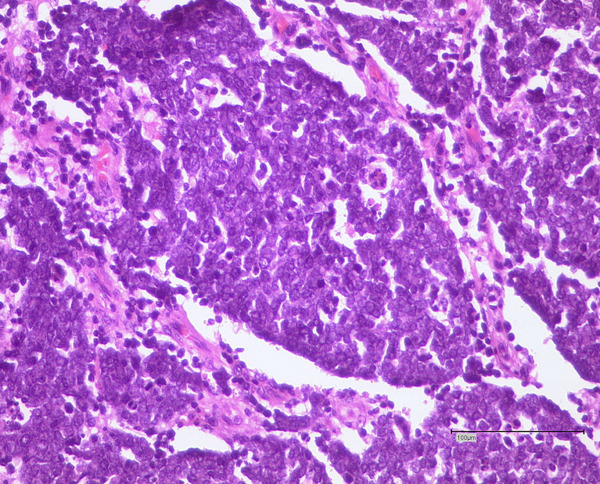
**Haematoxylin and eosin stain of the tumour of the elbow region**. Note the small cell characteristic of the Merkel cell carcinoma.

In contrast, the tumour from the elbow microscopically presented as a highly cellular small cell tumour lacking fascicular formations (Figure 2). Additional immunohistochemical analysis revealed punctual reaction of the vast majority of tumour cells for keratin MNF 116 with a cytoplasmatic distribution and a punctual perinuclear immune reaction for cytokeratin-20. The tumour was CD3, CD20 and LCA negative. A high proliferative activity index (Ki 67) of 70% was evident. The histological and immunohistochemical analysis verified the diagnosis of a Merkel cell carcinoma (pT_1_ pN_0_, pM_0_ R_0_).

The Merkel cell carcinoma was resected in total whereas a complete resection of the DFSP (pT_2b_, pN_0_, pM_0_, R_2_) with preservation of the extremity could not be achieved.

**Figure 3:**
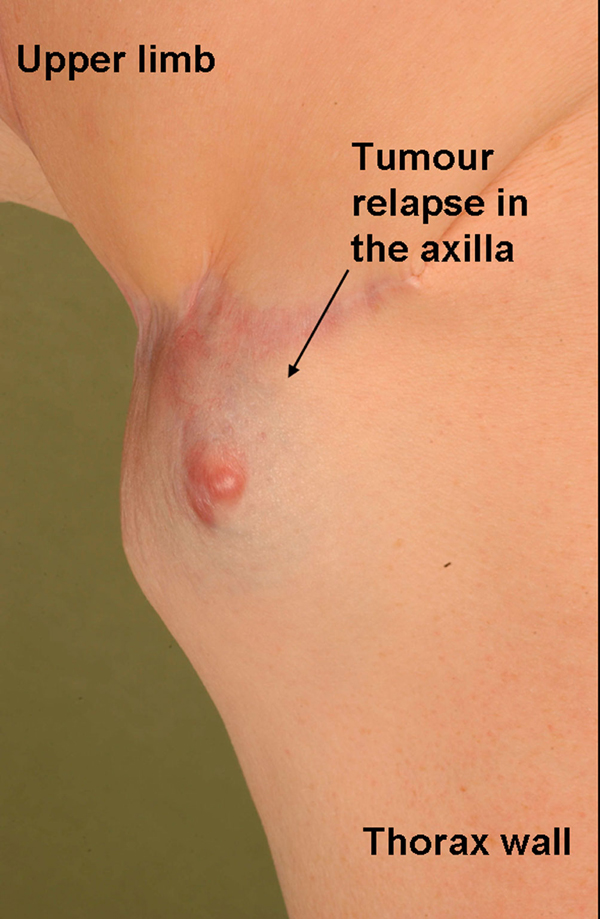
**Clinical presentation of the collision tumour relapse of the dermatofibrosarcoma protuberans and metastatic lesion of the Merkel cell carcinoma of the axilla**.

**Figure 4:**
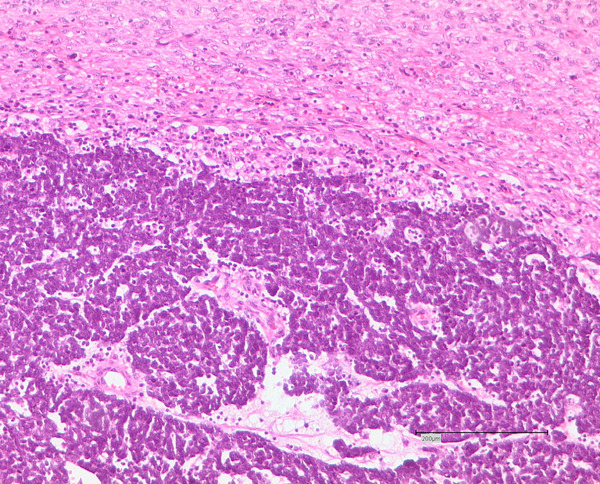
**Haematoxylin and eosin stain of the collision tumour of the metastatic Merkel cell tumour within the tumour relapse of the dermatofibrosarcoma protuberans**. Note the coexistence of a small cell tumour mass within a storiform, fascicular cell formation.

Six months after the surgery the patient suffered a synchronous local recurrence of both the DFSP and MCC (Figure 3). At this time the tumour specimen from the elbow histologically resembled the previously described MCC. The axillary specimen revealed an infiltration of a spindle cell-like tumour with a partial storiform, partial fascicular, growth pattern. Within this tumour, an area of up to 1.1 cm with a small cell tumour infiltrate, correlating with the described Merkel cell tumour mixed with a spindle cell tumour formation, was found. The histological findings displayed a collision tumour of a metastasis of the Merkel cell tumour (pT_3_, pN_0_, pM_1_) and the relapse tumour of DFSP (pT_2b_, pN_0_, pM0, R_2_) (Figure 4). Further clinical and radiological tumour staging demonstrated no further evidence of metastatic disease.

For local tumour control, the option of an interthoracoscapular amputation was discussed but not accepted by the patient. Therefore, palliative radiotherapy and chemotherapy with the tyrosine kinase inhibitor Imatinib was initiated.

## Discussion

Both DFSP and MCC are very rare malignancies. Even though the occurrence of multiple malignancies in the same anatomical site in organs such as the thyroid, breast and lymph nodes [4] have been described previously, the occurrence of synchronous colliding tumours remains an extremely uncommon condition with a very limited number of cases in the literature. Single cases of the simultaneous appearance of MCC with chronic lymphatic leukaemia [5] or rhabdomyosarcoma [6] have been described. To our knowledge this is the first reported case involving MCC and DFSP; in this patient, a haematogenous metastasis of a MCC within a relapse tumour of DFSP located in the axilla.

Even though the association between these two tumours in our patient is most likely coincidental, immunosuppression during a long course of previous tumour disease of the right upper extremity might be accountable for an increased risk of developing a secondary malignancy such as the MCC.

Clinical studies and animal experiments demonstrate the fact that malignant tumours lead to an altered immune status systemically, as well as locally, through changes in the microenvironment. Functional impairment of T-lymphocytes in addition to an accumulation of chronically activated myeloid suppressor cells and regulatory T-cells has been observed systemically. This may lead to a disabled tumour killing CD 8 CTL response and enhance cancer development through activation of other immune cells.

Changes in local microenvironment, for example an increase in certain pro-inflammatory cytokines and pro-angiogenic mediators such as interleukin 1 and 6 (IL1, IL6) tumour necrosis factor (TNF α) and vascular endothelial growth factor (VEGF), is one mechanism by which the tumour infiltrating leucocytes promote tumour growth, cell survival and an increase in tumour angiogenesis [7,8].

Many studies have confirmed an increase in certain matrix metalloproteinases (MMPs) altering tissue homeostasis through various biological pathways such as remodelling of the extracellular matrices, increase in cell-cell and cell-matrix adhesion molecules and thereby altering intracellular signalling pathways and often correlating with a poorer prognosis. But many of the mechanisms involved in tumour immunology and tumorigenesis, differentiation and dedifferentiation of cancer stem cells are still unknown, especially mechanisms involved in the synchronous appearance of malignant tumours [9].

For both malignancies described here, surgical resection of the tumour with clear surgical margins is the main choice of treatment. Both tumours are very rare neoplasms with a similar incidence of four cases or less per million per year. DFSP may affect patients of all ages but is rarely encountered during childhood and it mainly occurs in adults between 20 and 40 years of age. It is a tumour of the dermis and subcutis characterized by a local aggressive growth pattern. Its asymmetric growth and pseudopod-like extensions aggravate tumour resection and account for a high rate of local recurrence of up 60% [10]. A strong correlation between surgical margins and tumour free survival has been demonstrated. Invasion of the underlying fascia and muscle has been observed in patients with a long history of DFSP. In these cases wide local resection may be mutilating and in this case an interthoracoscapular resection would have been required. Fibrosarcomatous transformation of DFSP results in an increased risk of distant metastasis especially into the lungs and an even higher rate of local recurrence [11,12].

Radiotherapy has been successful in gaining local tumour control. DFSP has been found to be non-chemotherapy sensitive. Recently the tyrosine kinase inhibitor Imatinib has demonstrated an antitumorigenic effect with promising results for patients with metastatic disease as well as for local tumour control [13]. MCC on the other hand is a cutaneous small cell carcinoma of the elderly with a female predominance [1]. Impaired immune status due to viral infections or immunosuppression therapy has been linked to an increased risk of developing MCC [5]. In the case described here the patient had no history of immunosuppressive therapy and displayed no reasonable grounds to suspect HIV infection even though this was not confirmed by laboratory testing.

In the last 7 years, 12 patients with a MCC were treated in our clinic. Interestingly, four of these 12 patients had a history of an underlying previous malignancy and one additional patient suffered from chronic renal failure. Both conditions possibly lead to a compromised immune response. Compared to other extra pulmonary small cell cancers MCC appears to have a favourable prognosis but it remains an aggressive tumour with a frequent recurrence, early metastases and high mortality rates. In contrast to melanoma, which also represents a neuroendocrine tumour of the skin, tumour thickness seemingly is of no prognostic value. Regional lymph node status appears to influence local recurrence but not the overall prognosis [14]. MCC has the potential for haematogenous metastasis mainly affecting the lungs as well as lymphatic metastasis. Distant metastasis is the limiting prognostic factor. Similar to other small cell carcinomas radiotherapy and chemotherapy sensitivity has been described. Various clinical and histological parameters have been evaluated regarding their predictive prognostic value but findings remain contradictory [15].

## Conclusions

Both MCC and DFSP are uncommon tumour entities of elderly or immunocompromised patients. Collision tumours are extremely rare and have never been described for MCC and DFSP. In this patient the distant metastasis of the MCC is expected to be the limiting factor for survival.

## Abbreviations

DFSP: dermatofibrosarcoma protuberans; MCC: Merkel cell carcinoma; MMPs: matrix metalloproteinases; VEGF: vascular endothelial growth factor.

## Consent

Written informed consent was obtained from the patient for publication of this case report and any accompanying images. A copy of the written consent is available for review by the Editor-in-Chief of this journal.

## Competing interests

The authors declare that they have no competing interests.

## Authors' contributions

DT conceptualised the case report, gathered the data and wrote the manuscript. ML drafted and revised the manuscript and was involved in the primary surgery. JH gathered the clinical data and assisted with postoperative care of the patient. AD reviewed the literature and assisted in the follow up treatment of the patient. HH performed the primary surgery and took responsibility for the patient's care. HS conceptualised and supervised the process and he gave final approval for publication. CK assessed the histological specimens and drafted and revised the manuscript. All authors read and approved the final manuscript.
